# Prevalence and Associated Factors of Halitosis Among University Students in Guangxi, Southern China: A Cross-Sectional Study

**DOI:** 10.3290/j.ohpd.c_2326

**Published:** 2025-12-12

**Authors:** Haishan Zhang, Tingting Zhang, Xingguo Fu, Honglin Qin, Xiaochun Chen, Xiaojuan Zeng

**Affiliations:** a Haishan Zhang# PhD Student, Department of Preventive Dentistry, Guangxi Medical University College of Stomatology, Nanning, Guangxi, China. Performed clinical examination and assessment, analysed the data and drafted the manuscript.; b Tingting Zhang# Attending Physician, Guangxi Aiage Multidimensional Longevity Oral Technology Corporation Ltd., Nanning, Guangxi, China. Performed clinical examination and assessment.; c Xingguo Fu# Med Student, Department of Preventive Dentistry, Guangxi Medical University College of Stomatology, Nanning, Guangxi, China. Performed clinical examination and assessment.; d Honglin Qin Technician, Data Department of Aisheng Biotechnology Corporation Ltd., Nanning, Guangxi, China. Developed the analysis strategy and gave text suggestions.; e Xiaochun Chen Senior Engineer, Data Department of Aisheng Biotechnology Corporation Ltd., Nanning, Guangxi, China. Conceived and supervised the project, developed the analysis strategy.; f Xiaojuan Zeng Professor, Department of Preventive Dentistry, Guangxi Medical University College of Stomatology, Nanning, Guangxi, China. Conceived and supervised the project, critically reviewed the drafts and gave text suggestions, and approved the final manuscript. # These authors contributed equally to this work.

**Keywords:** associated factors, epidemiology, halitosis, oral health, young adults

## Abstract

**Purpose:**

This study aimed to investigate the prevalence of halitosis and explore associated factors among university students in Guangxi, Southern China.

**Materials and Methods:**

A cross-sectional study was conducted among university students aged 18–25 from 10 universities in Guangxi using multi-stage stratified random sampling. Face-to-face, paper-based questionnaires were administered to collect participants’ general information and data on self-reported halitosis. Organoleptic testing (OLT) was used as the clinical reference standard for diagnosing organoleptic halitosis. Standardised oral examinations were also conducted to evaluate participants’ oral health status. A bidirectional stepwise multivariable logistic regression analysis was performed to identify factors associated with self-reported and organoleptic halitosis.

**Results:**

Of the 1,377 participants, 34.9% self-reported halitosis, and 33.9% were diagnosed with organoleptic halitosis by the OLT. The agreement rate between the two methods was 65.4%, with a Cohen’s κ coefficient of 0.23, indicating fair concordance. In the final multivariate regression analysis, self-reported halitosis was significantly associated with self-perceived stress, dry mouth, food impaction, gingival bleeding, and respiratory tract diseases. In contrast, organoleptic halitosis was significantly associated with smoking, infrequent water intake, lack of daily tongue cleaning, and poor oral hygiene. The absence of regular dental scaling and a tongue coating score (TCS) ≥5 were common correlates of both self-reported and organoleptic halitosis.

**Conclusion:**

The prevalence of halitosis in this population based on the OLT was 33.9%. Early identification and patient education may help reduce the burden of halitosis and improve overall oral health.

Halitosis (also known as bad breath or oral malodour) is a clinical condition defined as unpleasant or offensive odours emanating from the oral cavity.^1,23^ It ranks as the third most common reason for dental consultations, following dental caries and periodontal disease, with a diagnosis rate of up to 65.9%.^11^ A meta-analysis estimated the global prevalence of halitosis to be 31.8% in the general population.^36^ According to the classification criteria established by the International Society for Breath Odour Research (ISBOR), halitosis is categorised into three clinical subtypes: genuine halitosis, pseudo-halitosis, and halitophobia.^20^ The latter two subtypes lack objectively detectable oral malodour and are mainly related to psychological factors.^13^ In most cases, the term ‘halitosis’ specifically refers to genuine halitosis. Based on the origin of the odour, genuine halitosis can be further subdivided into intraoral halitosis (IOH) and extraoral halitosis (EOH), with approximately 90% of cases attributed to IOH.^5,23^ Tongue coating and periodontal disease are considered the primary contributors to IOH.^35,45^ Other oral factors, such as deep carious lesions, exposed necrotic pulps, poor oral hygiene, food impaction, dry mouth, fixed orthodontic appliances, and mucosal ulcers, have also been implicated.^19,20,34^ In contrast, EOH is typically associated with systemic conditions, including respiratory tract infections, gastrointestinal diseases, diabetes, renal failure, and hepatic cirrhosis.^5,18,28^

The main malodorous components of halitosis-related gases are volatile sulfur compounds (VSCs), which are produced by Gram-negative anaerobes through the degradation of sulfur-containing amino acids.^37,45^ Among these, hydrogen sulphide (H_2_S), methyl mercaptan (CH_3_SH), and dimethyl sulphide ((CH_3_)_2_S) collectively account for over 90% of total VSCs.^23^ In addition to VSCs, amines (eg, cadaverine, putrescine), aromatic compounds (eg, indole, skatole, pyridine), and short-chain fatty acids (eg, acetic acid, propionic acid, butyric acid) also contribute to oral malodour, albeit to a lesser extent.^5^ Individuals with chronic halitosis often develop olfactory adaptation, making them unaware of their own oral malodour.^2,43^ They are frequently informed about the condition by partners, family members, or friends. This not only causes embarrassment but may also lead to a range of psychological effects, including anxiety, depression, low self-esteem, and social isolation, ultimately impairing oral health-related quality of life (OHRQoL).^33,39^

In clinical and epidemiological research, the three main methods for assessing halitosis are self-report questionnaires, organoleptic testing (OLT), and instrumental analysis.^11,14^ Self-report questionnaires, which reduce the time and cost of clinical examinations, are often employed as a preliminary screening tool to identify potential risk factors for halitosis in large populations.^1^ Although self-report questionnaires are simple and practical, OLT, based on Rosenberg’s criteria, remains the most widely used method and is currently regarded as the clinical gold standard for halitosis diagnosis.^12,31^ With standardised olfactory training, examiners can reliably assess the intensity of oral malodour. If precise identification and quantification of specific compounds are required, instrumental analysis is essential. For instance, the portable gas chromatograph Oral Chroma (Nissha FIS, Japan) can differentiate among the three major VSCs previously mentioned, while the sulphide monitor Halimeter (Interscan, USA) provides rapid quantification of total VSC concentrations.^22^

Current epidemiological studies on halitosis predominantly focus on middle-aged and elderly populations or children and adolescents. Young adults, especially university students undergoing the transition from adolescence to independent adulthood, exhibit a greater need for halitosis prevention and treatment. This is largely owing to their frequent social interactions and heightened concern for personal image.^8^ Despite the clinical relevance of halitosis in young adults, few studies have systematically estimated the prevalence of halitosis or explored related factors in this population. Existing data indicate that the prevalence of halitosis among young adults ranges from 15% to 55.97%.^4,7,10^ However, many of these studies rely on convenience samples or do not include OLT performed by dental professionals, which may compromise the reliability of their findings.

Therefore, this study aims to investigate the prevalence of halitosis and associated factors among young adults in Guangxi, represented by university students, using self-reporting and OLT methods. As the first epidemiological study on halitosis in young adults in Guangxi, this research will provide a theoretical foundation for developing targeted halitosis management strategies.

## MATERIALS AND METHODS

### Ethics Statement

This study was conducted in accordance with the ethical principles of the Declaration of Helsinki and was approved by the Ethics Committee of Guangxi Medical University (Approval No. 2024 KY0226). Written informed consent was obtained from all participants.

### Participants and Study Design

The study population comprised university students aged 18–25 years in Guangxi, China. Data was collected from 1 September to 31 December 2024. The required sample size was calculated using the single population proportion formula^10^:

,

assuming a prevalence of 50% (no previous study in Guangxi), a 95% confidence level (Z = 1.96), and a margin of error (E) of 3%. After adjusting for a 20% non-response rate, the final sample size was determined to be 1,335 participants. A multi-stage stratified random sampling method was employed. First, the 14 cities in Guangxi were stratified based on educational resources into two groups: Nanning, which hosts nearly 50% of the region’s universities, and the remaining 13 cities. Three universities were randomly selected from Nanning, and seven cities were randomly chosen from the latter group, with one university selected from each, resulting in a total of 10 universities. Second, students in the target universities were stratified by academic year into freshman, sophomore, and junior groups (seniors excluded due to off-campus internships). Finally, one class from each eligible grade was randomly chosen, and all students were invited to participate.

Participants received written instructions one week prior to their scheduled appointment. They were asked to: (1) avoid consuming pungent foods (eg, onions, garlic), smoking, or drinking alcohol for 48 h before the assessment; (2) refrain from using oral rinses, breath fresheners, chewing gum, mints, or scented personal care products on the morning of the assessment; (3) finish toothbrushing and eating breakfast at least 2 h before the scheduled assessment time. All halitosis assessments were conducted in the morning between 8:00 a.m. and 11:30 a.m.^1,26,43^ Exclusion criteria included antibiotic use or active periodontal therapy within the past 30 days.^11^ On the day of the appointment, participants were asked to reconfirm their adherence to the pre-assessment instructions during registration. Those who failed to comply were excluded from the study. To avoid verification bias, the questionnaire was completed prior to the OLT.

### Questionnaire

A semi-structured questionnaire was developed based on the World Health Organization (WHO)’s *Basic Methods for Oral Health Surveys* (5th edition), with adaptations from previous studies.^6,9,11,14^ It was pilot-tested with 20 volunteers who did not participate in the main study to refine question wording for clarity and consistency before final use. The paper-based questionnaire was administered face-to-face by trained investigators. It collected information on participants’ demographic characteristics, self-reported health status, lifestyle and dietary behaviours, and oral hygiene practices, including detailed data on the frequency and amount of behavioural factors relevant to halitosis. The questionnaire was designed to be completed within 10 min. Self-reported halitosis was assessed using a single-item question: ‘Do you think you have bad breath?’ (Yes/No).^44^ Additional information on participants’ medication use and the time periods of halitosis occurrence was also recorded. To ensure sufficient sample sizes for meaningful analysis and to avoid sparse subgroup comparisons, certain response categories were merged as needed during data processing.

### Organoleptic Assessment

The OLT was conducted before oral clinical examinations to avoid potential interference with assessment accuracy due to gingival bleeding caused by periodontal probing. A panel of three experienced dentists who were blinded to all other data performed the OLT in a standardised manner.^16^ These examiners had been trained in distinguishing odours using the Smell Identification Test (Sensonics, Haddon Heights, NJ, USA). A 50 cm × 50 cm opaque privacy screen with a central hole was placed between participants and examiners. During the test, participants were instructed to sit upright with their lips closed for 1 min, followed by nasal breathing and gentle exhalation through a disposable plastic tube (1 cm in diameter and 20 cm in length). Two examiners independently assessed the exhaled breath at the opposite end of the tube and assigned organoleptic scores (OS) following the Rosenberg organoleptic scale^31^: 0 = no odour, 1 = barely noticeable odour, 2 = slight malodour, 3 = moderate malodour, 4 = strong malodour, and 5 = severe malodour. The final OS was recorded if the two scores were consistent. In cases of disagreement, a third examiner performed an independent assessment. If the third score matched either of the first two, that score was adopted. If all three scores differed, the final OS was determined by consensus through joint discussion. To prevent olfactory fatigue from repeated odour exposure, a 5-min rest interval was implemented between consecutive tests.^6^ In this study, OLT served as the clinical diagnostic tool for halitosis, and participants were classified as having organoleptic halitosis when their OS ≥2.^27^ Inter-rater reliability of the OLT was assessed in the pilot study mentioned above (n = 20), with a Fleiss’ κ coefficient of 0.74 based on independent ratings from three examiners.

### Oral Clinical Examination

Two experienced dentists who were trained but not involved in the organoleptic assessment examined the intraoral conditions of the participants. All examinations were conducted under artificial lighting, using a Community Periodontal Index (CPI) probe and a mouth mirror for inspection, in conjunction with probing. Oral hygiene status was assessed using the Oral Hygiene Index-Simplified (OHI-S), calculated as the sum of the Debris Index-Simplified (DI-S) and Calculus Index-Simplified (CI-S). DI-S and CI-S were recorded by examining the labial/buccal surfaces of teeth 16, 11, 26, and 31, and the lingual/palatal surfaces of teeth 36 and 46. Caries status was evaluated with the Decayed, Missing, and Filled Teeth (DMFT) index. The Gingival Index (GI) measured gingival inflammation at four sites on six index teeth (16, 12, 24, 32, 36, 44). Periodontal probing depth (PPD) was measured at four sites of six index teeth (16, 11, 26, 31, 36, 46), with the maximum PPD value recorded. Tongue coating status was classified using the Kojima scoring criteria.^1,41^ The tongue coating score (TCS) was calculated by multiplying the area score by the thickness score, with TCS ranging from 0 to 9.30. Oral mucosal conditions and denture status were also recorded. To assess inter-examiner reliability, a randomly selected 10% of participants were re-examined. Cohen’s κ coefficients for all oral clinical parameters ranged from 0.82 to 0.90.

### Statistical Analysis

The data were entered using EpiData version 3.1 (EpiData Association, Odense, Denmark) and analysed with IBM SPSS Statistics version 22 (IBM, Armonk, NY, USA). Descriptive statistics were used to summarise participant characteristics. Categorical variables were presented as frequencies and percentages, while continuous variables were expressed as means ± standard deviations. Group comparisons were performed using Student’s t-test for continuous variables, and the Chi-square test or Fisher’s exact test for categorical variables. Variables significantly associated with self-reported or organoleptic halitosis in bivariate analysis were entered into multivariable logistic regression models using bidirectional stepwise selection to identify independent correlates. Adjusted odds ratios (ORs) and 95% confidence intervals (CIs) were reported. Multicollinearity was assessed using the Generalised Variance Inflation Factor (GVIF), with values calculated for each independent variable. A two-tailed P-value <0.05 was considered statistically significant.

## RESULTS

Of the 1,523 university students invited, 101 were excluded for not providing informed consent, 24 due to incomplete questionnaires, and 21 for recent antibiotic use. A total of 1,377 participants (response rate: 90.4%) were included in the final analysis. The mean age of participants was 19.8 ± 1.2 years, with 61.0% females and 39.0% males. No significant age difference was observed between genders (P = 0.103).

**Fig 1:**
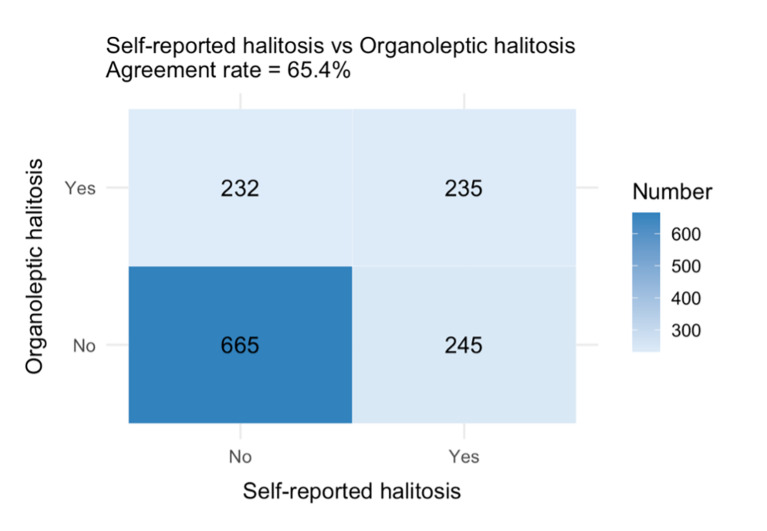

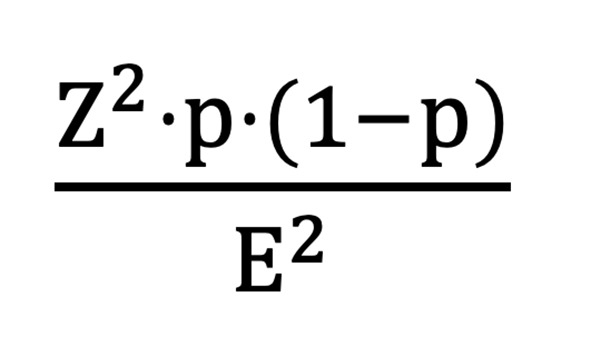
The agreement rate between self-reported halitosis and organoleptic halitosis.

Among all participants, 34.9% self-reported experiencing halitosis, while 33.9% were identified with an OS ≥2 through OLT. The agreement rate between the self-reporting and OLT was 65.4%, with a Cohen’s κ coefficient of 0.23 (95% CI: 0.18–0.29) (Fig 1). Using OLT as the reference standard, self-reporting showed a sensitivity of 0.503 and specificity of 0.731. Of those who self-reported halitosis (n = 480), 72.5% perceived their condition as most severe in the morning, 14.2% in the afternoon, 8.5% in the evening, and 4.8% experienced severe halitosis throughout the day. According to the OLT results, 7.2% of participants received a score of 0, 55.5% scored 1, 29.0% scored 2, 7.5% scored 3, and 0.8% scored 4. No participant had an OS of 5.

As shown in Table 1, no significant associations were found between self-reported halitosis and gender (P = 0.509), age (P = 0.539), grade (P = 0.558), academic major (P = 0.385), or BMI (P = 0.235). In contrast, organoleptic halitosis was significantly more prevalent among males than females (37.6% vs 31.5%, P = 0.024), and other demographic variables also showed no significant associations (P >0.05).

**Table 1:** Relationships between halitosis and participants’ demographic characteristics

Variables	Self-reported halitosis	P-value	Organoleptic halitosis	P-value
Yes	No	Yes	No
n (%)	n (%)	n (%)	n (%)
**Gender**			0.509			**0.024**
Male	181 (33.7%)	356 (66.3%)		202 (37.6%)	335 (62.4%)	
Female	299 (35.6%)	541 (64.4%)		265 (31.5%)	575 (68.5%)	
**Age**			0.539			0.443
<20 years	351 (34.3%)	671 (65.7%)		353 (34.5%)	669 (65.5%)	
≥20 years	129 (36.3%)	226 (63.7%)		114 (32.1%)	241 (67.9%)	
**Grade**			0.558			0.159
1^st^	170 (36.2%)	300 (63.8%)		169 (36.0%)	301 (64.0%)	
2^nd^	167 (35.4%)	305 (64.6%)		166 (35.2%)	306 (64.8%)	
3^rd^	143 (32.9%)	292 (67.1%)		132 (30.3%)	303 (69.7%)	
**Academic major**			0.385			0.390
Non-health-related	445 (34.5%)	843 (65.5%)		440 (34.2%)	848 (65.8%)	
Health-related	35 (39.3%)	54 (60.7%)		27 (30.3%)	62 (69.7%)	
**BMI**			0.235			0.569
Healthy	287 (35.0%)	532 (65.0%)		283 (34.6%)	536 (65.4%)	
Underweight	122 (32.4%)	254 (67.6%)		119 (31.6%)	257 (68.4%)	
Overweight	53 (36.6%)	92 (63.4%)		54 (37.2%)	91 (62.8%)	
Obese	18 (48.6%)	19 (51.4%)		11 (29.7%)	26 (70.3%)	
**Organoleptic halitosis**	**<0.001**			
No	245 (26.9%)	665 (73.1%)				
Yes	235 (50.3%)	232 (49.7%)				
**Self-reported halitosis**					**<0.001**
No				232 (25.9%)	665 (74.1%)	
Yes				235 (49.0%)	245 (51.0%)	
P-values were determined by Chi-square test.

Table 2 presents the associations between halitosis and self-reported health status. Self-reported halitosis was significantly associated with stress perception (P <0.001), dry mouth (P <0.001), food impaction (P <0.001), gingival bleeding (P <0.001), respiratory diseases (P = 0.038), and gastrointestinal diseases (P = 0.044). By comparison, organoleptic halitosis showed a significant association only with gingival bleeding (P = 0.003).

**Table 2:** Relationships between halitosis and participants’ self-reported health status

Variables	Self-reported halitosis	P-value	Organoleptic halitosis	P-value
Yes	No	Yes	No
n (%)	n (%)	n (%)	n (%)
**Stress perception**			**<0.001**			0.056
No	263 (29.5%)	629 (70.5%)		286 (32.1%)	606 (67.9%)	
Yes	217 (44.7%)	268 (55.3%)		181 (37.3%)	304 (62.7%)	
**Dry mouth**			**<0.001**			0.158
No	161 (24.0%)	511 (76.0%)		215 (32.0%)	457 (68.0%)	
Yes	319 (45.2%)	386 (54.8%)		252 (35.7%)	453 (64.3%)	
**Food impaction**			**<0.001**			0.074
No	180 (24.9%)	543 (75.1%)		229 (31.7%)	494 (68.3%)	
Yes	300 (45.9%)	354 (54.1%)		238 (36.4%)	416 (63.6%)	
**Gingival bleeding**			**<0.001**			**0.003**
No	327 (31.3%)	719 (68.7%)		332 (31.7%)	714 (68.3%)	
Yes	153 (46.2%)	178 (53.8%)		135 (40.8%)	196 (59.2%)	
**Respiratory tract diseases**		**0.038**			0.946
No	420 (33.9%)	818 (66.1%)		419 (33.8%)	819 (66.2%)	
Yes	60 (43.2%)	79 (56.8%)		48 (34.5%)	91 (65.5%)	
**Gastrointestinal diseases**			**0.044**			0.588
No	455 (34.3%)	871 (65.7%)		452 (34.1%)	874 (65.9%)	
Yes	25 (49.0%)	26 (51.0%)		15 (29.4%)	36 (70.6%)	
**Metabolic diseases**			1.000#			1.000#
No	479 (34.9%)	894 (65.1%)		466 (33.9%)	907 (66.1%)	
Yes	1 (25.0%)	3 (75.0%)		1 (25.0%)	3 (75.0%)	
**Hepatic diseases**			0.770#			0.790#
No	480 (34.9%)	895 (65.1%)		467 (34.0%)	908 (66.0%)	
Yes	0 (0%)	2 (100%)		0 (0%)	2 (100%)	
**Renal diseases**			1.000#			1.000#
No	480 (34.9%)	896 (65.1%)		467 (33.9%)	909 (66.1%)	
Yes	0 (0%)	1 (100%)		0 (0%)	1 (100%)	
P-values were determined by Chi-square test and ^#^Fisher exact test.

According to Table 3, participants who reported daily consumption of sweet foods (40.5% vs 31.0%, P = 0.043) or pungent foods (46.3% vs 31.6%, P = 0.003) had a significantly higher prevalence of self-reported halitosis compared to those who did not. In terms of organoleptic halitosis, prevalence was notably higher among current smokers (47.4% vs 32.9%, P = 0.006) and alcohol drinkers (37.4% vs 31.7%, P = 0.032), while frequent water intake (>8 times/day) was significantly associated with a lower prevalence (24.1% vs 42.3%, P = 0.001).

**Table 3:** Relationships between halitosis and participants’ lifestyle and dietary behaviours

Variables	Self-reported halitosis	P-value	Organoleptic halitosis	P-value
Yes	No	Yes	No
n (%)	n (%)	n (%)	n (%)
**Smoking**			0.595			**0.006**
Non-/former smokers	444 (34.6%)	838 (65.4%)		422 (32.9%)	860 (67.1%)	
Current smokers	36 (37.9%)	59 (62.1%)		45 (47.4%)	50 (52.6%)	
**Alcohol consumption**			0.261			**0.032**
Non-/former drinkers	303 (36.1%)	537 (63.9%)		266 (31.7%)	574 (68.3%)	
Current drinkers	177 (33.0%)	360 (67.0%)		201 (37.4%)	336 (62.6%)	
**Sweet foods intake**			**0.043**			0.567
Occasionally/Never	134 (31.0%)	298 (69.0%)		155 (35.9%)	277 (64.1%)	
Weekly	246 (35.2%)	452 (64.8%)		229 (32.8%)	469 (67.2%)	
Daily	100 (40.5%)	147 (59.5%)		83 (33.6%)	164 (66.4%)	
**Pungent foods intake**			**0.003**			0.916
Occasionally/Never	260 (31.6%)	564 (68.4%)		283 (34.3%)	541 (65.7%)	
Weekly	182 (38.6%)	289 (61.4%)		157 (33.3%)	314 (66.7%)	
Daily	38 (46.3%)	44 (53.7%)		27 (32.9%)	55 (67.1%)	
**Coffee intake**			0.207			0.900
Occasionally/Never	385 (33.9%)	751 (66.1%)		383 (33.7%)	753 (66.3%)	
Weekly	83 (40.3%)	123 (59.7%)		71 (34.5%)	135 (65.5%)	
Daily	12 (34.3%)	23 (65.7%)		13 (37.1%)	22 (62.9%)	
**Tea intake**			0.496			0.121
Occasionally/Never	355 (34.5%)	674 (65.5%)		340 (33.0%)	689 (67.0%)	
Weekly	106 (34.9%)	198 (65.1%)		106 (34.9%)	198 (65.1%)	
Daily	19 (43.2%)	25 (56.8%)		21 (47.7%)	23 (52.3%)	
**Daily water intake**			0.739			0.135
≥3 L	9 (31.0%)	20 (69.0%)		13 (44.8%)	16 (55.2%)	
2–3 L	48 (35.3%)	88 (64.7%)		39 (28.7%)	97 (71.3%)	
1–2 L	269 (33.9%)	525 (66.1%)		260 (32.7%)	534 (67.3%)	
<1 L	154 (36.8%)	264 (63.2%)		155 (37.1%)	263 (62.9%)	
**Water intake frequency**			0.145			**0.001**
>8 times/day	85 (36.6%)	147 (63.4%)		56 (24.1%)	176 (75.9%)	
6–8 times/day	136 (30.6%)	309 (69.4%)		144 (32.4%)	301 (67.6%)	
3–5 times/day	217 (36.8%)	372 (63.2%)		220 (37.4%)	369 (62.6%)	
1–2 times/day	42 (37.8%)	69 (62.2%)		47 (42.3%)	64 (57.7%)	
P-values were determined by Chi-square test.

Table 4 shows the relationship between halitosis and oral hygiene practices. Regular dental scaling was significantly associated with both self-reported (P = 0.002) and organoleptic halitosis (P = 0.001). For organoleptic halitosis, tooth brushing frequency (P = 0.004) and daily tongue cleaning (P <0.001) were also significantly associated.

**Table 4:** Relationships between halitosis and participants’ oral hygiene practices

Variables	Self-reported halitosis	P-value	Organoleptic halitosis	P-value
Yes	No	Yes	No
n (%)	n (%)	n (%)	n (%)
**Toothbrushing**			0.523			**0.004**
≥2 times/day	377 (34.4%)	719 (65.6%)		351 (32.0%)	745 (68.0%)	
<2 times/day	103 (36.7%)	178 (63.3%)		116 (41.3%)	165 (58.7%)	
**Floss daily**			0.148			0.119
Yes	107 (31.5%)	233 (68.5%)		103 (30.3%)	237 (69.7%)	
No	373 (36.0%)	664 (64.0%)		364 (35.1%)	673 (64.9%)	
**Mouthrinse use**			0.402			0.067
Yes	64 (32.0%)	136 (68.0%)		56 (28%)	144 (72%)	
No	416 (35.3%)	761 (64.7%)		411 (34.9%)	766 (65.1%)	
**Gum chewing**			0.511			0.464
Yes	25 (30.9%)	56 (69.1%)		31 (38.3%)	50 (61.7%)	
No	455 (35.1%)	841 (64.9%)		436 (33.6%)	860 (66.4%)	
**Tongue cleaning daily**			0.094			**<0.001**
Yes	115 (31.2%)	254 (68.8%)		86 (23.3%)	283 (76.7%)	
No	365 (36.2%)	643 (63.8%)		381 (37.8%)	627 (62.2%)	
**Regular dental scaling**			**0.002**			**0.001**
Yes	88 (27.3%)	234 (72.7%)		84 (26.1%)	238 (73.9%)	
No	392 (37.2%)	663 (62.8%)		383 (36.3%)	672 (63.7%)	
P-values were determined by Chi-square test.

Analysis of oral clinical examination indicators (Table 5) indicated that both self-reported and organoleptic halitosis were significantly associated with GI and TCS (P <0.05). Additionally, organoleptic halitosis was also significantly associated with OHI-S (P <0.001). No significant associations were observed between DMFT, PPD, or denture prosthesis status and either self-reported or organoleptic halitosis (P >0.05).

**Table 5:** Relationships between halitosis and participants’ oral clinical examination indicators

Variables	Self-reported halitosis	P-value	Organoleptic halitosis	P-value
Yes	No	Yes	No
n (%)	n (%)	n (%)	n (%)
**OHI-S**			0.119			**<0.001**
Good	317 (33.6%)	627 (66.4%)		275 (29.1%)	669 (70.9%)	
Fair	133 (36.2%)	234 (63.8%)		160 (43.6%)	207 (56.4%)	
Poor	30 (45.5%)	36 (54.5%)		32 (48.5%)	34 (51.5%)	
**DMFT**			0.432			0.550
0	123 (33.1%)	249 (66.9%)		121 (32.5%)	251 (67.5%)	
≥1	357 (35.5%)	648 (64.5%)		346 (34.4%)	659 (65.6%)	
**GI**			**0.023**			**0.005**
<1	404 (33.5%)	801 (66.5%)		391 (32.4%)	814 (67.6%)	
1–1.5	55 (44.0%)	70 (56.0%)		52 (41.6%)	73 (58.4%)	
>1.5	21 (44.7%)	26 (55.3%)		24 (51.1%)	23 (48.9%)	
**PPD**			1.000			0.534
<3.5 mm	469 (34.9%)	876 (65.1%)		454 (33.8%)	891 (66.2%)	
≥3.5 mm	11 (34.4%)	21 (65.6%)		13 (40.6%)	19 (59.4%)	
**Denture prosthesis**			0.176			0.070
No	412 (34.2%)	794 (65.8%)		398 (33.0%)	808 (67.0%)	
Yes	68 (39.8%)	103 (60.2%)		69 (40.4%)	102 (59.6%)	
**TCS**			**<0.001**			**<0.001**
0–1	152 (31.7%)	327 (68.3%)		102 (21.3%)	377 (78.7%)	
2–4	257 (33.1%)	519 (66.9%)		296 (38.1%)	480 (61.9%)	
≥5	71 (58.2%)	51 (41.8%)		69 (56.6%)	53 (43.4%)	
P-values were determined by Chi-square test.

Variables that showed significant associations with halitosis in bivariate analyses were further entered into multivariable logistic regression models, which identified distinct correlates of self-reported and organoleptic halitosis. All GVIF^(1/(2·Df)) values were below 1.04 in each model, indicating no notable multicollinearity. For self-reported halitosis, significant correlates included stress perception (adjusted OR = 1.340), dry mouth (adjusted OR = 2.076), food impaction (adjusted OR = 2.027), gingival bleeding (adjusted OR = 1.374), respiratory tract diseases (adjusted OR = 1.486), absence of regular dental scaling (adjusted OR = 1.408) and TCS ≥ 5 (adjusted OR = 2.743) (Table 6). With regard to organoleptic halitosis, significant correlates included current smokers (adjusted OR = 1.978), infrequent water intake (adjusted OR = 1.874), lack of daily tongue cleaning (adjusted OR = 1.551), absence of regular dental scaling (adjusted OR = 1.447), poor oral hygiene (adjusted OR = 1.571), and TCS ≥ 5 (adjusted OR = 3.326) (Table 7).

**Table 6:** Multivariable logistic regression analysis of correlates of self-reported halitosis

Variables	Crude OR (95% CI)	P-value	Adjusted OR (95% CI)	P-value
**Stress perception**				
No	Ref		Ref	
Yes	1.937 (1.539, 2.437)	<0.001	1.340 (1.043, 1.722)	**0.022**
**Dry mouth**				
No	Ref		Ref	
Yes	2.623 (2.082, 3.305)	<0.001	2.076 (1.620, 2.661)	**<0.001**
**Food impaction**				
No	Ref		Ref	
Yes	2.556 (2.035, 3.212)	<0.001	2.027 (1.588, 2.587)	**<0.001**
**Gingival bleeding**				
No	Ref		Ref	
Yes	1.890 (1.468, 2.433)	<0.001	1.374 (1.047, 1.804)	**0.022**
**Respiratory tract diseases**				
No	Ref		Ref	
Yes	1.479 (1.036, 2.111)	0.031	1.486 (1.013, 2.180)	**0.043**
**Gastrointestinal diseases**				
No	Ref		NI	
Yes	1.841 (1.051, 3.224)	0.033		
**Pungent foods intake**				
Occasionally/Never	Ref		Ref	
Weekly	1.366 (1.078, 1.731)	0.010	1.265 (0.983, 1.628)	0.068
Daily	1.873 (1.185, 2.962)	0.007	1.474 (0.902, 2.411)	0.122
**Regular dental scaling**				
Yes	Ref		Ref	
No	1.572 (1.194, 2.070)	0.001	1.408 (1.048, 1.890)	**0.023**
**GI**				
<1	Ref		NI	
1–1.5	1.558 (1.073, 2.262)	0.020		
>1.5	1.601 (0.890, 2.881)	0.116		
**TCS**				
0–1	Ref		Ref	
2–4	1.065 (0.835, 1.359)	0.611	1.072 (0.828, 1.388)	0.598
≥5	2.995 (1.992, 4.504)	<0.001	2.743 (1.779, 4.229)	**<0.001**
OR: odd ratio; Ref: reference category; NI: not included in the model.

**Table 7:** Multivariable logistic regression analysis of correlates of organoleptic halitosis

Variables	Crude OR (95% CI)	P-value	Adjusted OR (95% CI)	P-value
**Gender**				
Male	Ref		NI	
Female	0.764 (0.609, 0.959)	0.020		
**Gingival bleeding**				
No	Ref		Ref	
Yes	1.481 (1.148, 1.911)	0.003	1.265 (0.965, 1.660)	0.089
**Smoking**				
Non- and past-smokers	Ref		Ref	
Current smokers	1.834 (1.206, 2.789)	0.005	1.978 (1.279, 3.060)	**0.002**
**Alcohol consumption**				
Non- and past-drinkers	Ref		NI	
Current drinkers	1.291 (1.028, 1.620)	0.028		
**Water intake frequency**				
>8 times/day	Ref		Ref	
6–8 times/day	1.504 (1.049, 2.156)	0.027	1.442 (0.991, 2.098)	0.056
3–5 times/day	1.874 (1.328, 2.643)	<0.001	1.751 (1.224, 2.505)	**0.002**
1–2 times/day	2.308 (1.426, 3.737)	0.001	1.874 (1.128, 3.111)	**0.015**
**Toothbrushing**				
≥2 times/day	Ref		NI	
<2 times/day	1.492 (1.140, 1.953)	0.004		
**Tongue cleaning daily**				
Yes	Ref		Ref	
No	2.000 (1.522, 2.627)	<0.001	1.551 (1.163, 2.070)	**0.003**
**Regular dental scaling**				
Yes	Ref		Ref	
No	1.615 (1.222, 2.134)	0.001	1.447 (1.081, 1.936)	**0.013**
**OHI-S**				
Good	Ref		Ref	
Fair	1.880 (1.465, 2.413)	<0.001	1.330 (0.776, 2.280)	0.300
Poor	2.290 (1.385, 3.785)	0.001	1.571 (1.209, 2.043)	**0.001**
**GI**				
<1	Ref		NI	
1–1.5	1.483 (1.019, 2.159)	0.040		
>1.5	2.172 (1.211, 3.897)	0.009		
**TCS**				
0–1	Ref		Ref	
2–4	2.279 (1.753, 2.963)	<0.001	1.948 (1.484, 2.556)	**<0.001**
≥5	4.812 (3.163, 7.320)	<0.001	3.326 (2.119, 5.221)	**<0.001**
OR: odd ratio; Ref: reference category; NI: not included in the model.

## DISCUSSION

Halitosis is prevalent worldwide. It is not only a common oral health problem but also a potential threat to psychosocial well-being. Previous studies have indicated that individuals with halitosis tend to exhibit more introverted personality traits and are more likely to experience social avoidance compared to those without the condition.^17,42^ Although halitosis has been widely researched, its reported prevalence varies considerably across regions and populations. Among school-aged children, Ueno et al^38^ reported a 44.9% prevalence of halitosis in Japan, while a much lower rate was observed in Brazil (17.3%).^40^ Conversely, in the adolescent population, the condition was more common in Brazil (39.7%)^24^ than in Japan (26.0%).^29^ For adults, the prevalence has been reported to range from 31.5% to 53.5% in different countries.^1,6^ Among the elderly in Thailand, over 60% were affected, which may result from deteriorating periodontal conditions and a high burden of systemic diseases.^32^ Overall, the occurrence of halitosis appears to increase with age, though age alone does not fully account for these disparities. Differences in ethnic and cultural backgrounds, as well as the heterogeneity of diagnostic criteria and assessment methods, could also influence the estimated prevalence of halitosis. Epidemiological research on halitosis among young adults remains limited.

In this study, 34.9% of university students self-reported having halitosis, which is lower than the 55.97% self-reported prevalence in a convenience sample of university students from Dhaka, Bangladesh.^10^ Another study focusing on dental students found a self-reported prevalence of only 21.8% (0.4% persistent cases), possibly due to their better oral hygiene practices compared to non-dental peers.^25^ With respect to organoleptic halitosis assessed by the OLT, the prevalence of 33.9% in our study was higher than the 24.9% observed in Swiss young adults,^7^ but comparable to the 31.2% in young adults from Dunedin, New Zealand.^44^ The slightly higher prevalence of self-reported halitosis (34.9%) compared to organoleptic halitosis (33.9%) may partly reflect young adults’ greater concern about oral health and self-image.^26^ Although the prevalence estimates from the two methods were relatively close, the overall agreement rate was moderate at 64.6%, while the Cohen’s κ coefficient of 0.23 indicated only fair concordance beyond random chance. Using the OLT results as the reference standard to determine the presence or absence of oral malodour, self-reporting showed a sensitivity of 0.503 and specificity of 0.731. This means that 50.3% of participants with genuine halitosis accurately reported their condition, while 73.1% of those without halitosis correctly reported its absence. In other words, nearly half (49.7%) of the participants with genuine halitosis failed to recognise or report it (false negatives), and over a quarter (26.9%) reported having oral malodour despite no clinical evidence (false positives). A population-based study in Brazil also found that self-reporting is relatively effective in identifying individuals without halitosis, but less accurate for those who actually have the condition.^14^ Our findings further highlight the limitations of relying solely on self-reporting to estimate the prevalence of halitosis in populations, as it is potentially influenced by pseudo-halitosis and halitophobia, along with behavioural tendencies like social desirability bias.

In the bivariate analysis, the proportion of self-reported halitosis was higher among females than males, whereas organoleptic halitosis showed the opposite trend with a statistically significant gender association. This discrepancy could be attributed to physiological factors, such as menstrual cycle regularity and hormonal fluctuations, which are known to affect females’ odour perception.^3,21^ However, most epidemiological studies have not identified a consistent association between gender and halitosis prevalence.^6,38^ In our final adjusted models, gender was not significantly associated with either self-reported or organoleptic halitosis. Notably, correlates of self-reported halitosis were primarily subjective indicators related to individuals’ self-perception, whereas organoleptic halitosis was more strongly associated with objective factors such as daily behaviours, oral hygiene practices, and clinical examination findings.

Participants reporting symptoms of dry mouth, food impaction, and gum bleeding had significantly higher odds of self-reported halitosis, consistent with the findings of Faria et al.^14^ Specifically, Dry mouth may impair the oral self-cleaning function by reducing saliva flow, thereby facilitating the proliferation of Gram-negative anaerobes.^44^ Food impaction provides additional metabolic substrates for these bacteria, further exacerbating the production of VSC. Gingival bleeding, a clinical manifestation of gingival inflammation, is closely linked to periodontal health. It has been well established that individuals with periodontal disease are more likely to have oral malodour.^1,35^ Moreover, the breakdown of blood components can release sulfur-containing amino acids, which serve as precursors for VSCs production.^11^ These findings underscore the importance of periodontal care in mitigating the risk of halitosis. However, it should be noted that the participants’ subjective perceptions may not always align with the actual oral health status. In contrast to self-reported halitosis, organoleptic halitosis more accurately reflects the objective presence of oral malodour. Smoking is classified as an extrinsic cause of halitosis rather than an intra-oral or extraoral endogenous factor.^27,30^ Although the proportion of current smokers was quite low in the present study, and all participants were required to abstain from smoking for 48 h before the assessment, smoking behaviour remained significantly associated with higher odds of organoleptic halitosis. One possible explanation for this association is long-term smoking-induced alterations in the oral environment. Nicotine and other tobacco constituents may perpetuate dry mouth symptoms by suppressing salivary gland function, while tobacco combustion products could alter oral pH and disrupt microbial homeostasis.^15^ Interestingly, we found that the frequency of daily water intake, rather than the total volume consumed, was associated with a protective effect against halitosis, which has rarely been reported in previous studies.

It is noteworthy that regular dental scaling and TCS were retained in the final regression models for both self-reported and organoleptic halitosis, suggesting a consistent contribution of routine professional dental care and tongue coating accumulation to subjective and objective assessments of oral malodour. Individuals who undergo regular dental scaling generally demonstrate greater oral health awareness and are more likely to seek professional intervention for potential oral problems, which may, to some extent, reduce the risk of halitosis. Through the mechanical removal of subgingival plaque and dental calculus, this practice not only eliminates potential etiological factors of halitosis but also helps alleviate patients’ undue concern regarding perceived oral malodour. On the other hand, tongue coating has been extensively recognised as a major source of halitosis.^11,20,26^ In a study among young adults in Switzerland, tongue coating was the only influencing factor for higher OS and VSC levels.^7^ The deep fissures, lingual crypts, and papillary structures on the tongue surface provide an ideal environment for the retention of desquamated epithelial cells and food debris, which are not easily flushed away by saliva.^5,43^ In addition, these anatomical features also facilitate bacterial colonisation. The colonising microbes subsequently degrade substrates and produce malodorous compounds that directly lead to halitosis. Among school-aged children, tongue coating has also been reported as a potential risk factor for halitosis.^38^ However, no significant association was observed between halitosis and tongue cleaning practices in this population, possibly explained by the fact that children have not yet mastered proper tongue cleaning techniques, making it difficult to effectively remove tongue coating.

Our study also identified respiratory diseases as a correlate of self-reported halitosis. Rhinitis was the most commonly reported condition among participants with self-reported respiratory diseases. In such cases, halitosis may result from the accumulation of VSCs in the paranasal sinuses, leading to the emission of malodorous gases from both the oral and nasal cavities.^5^ These patients require a comprehensive treatment approach to improve breath odour. As the study population comprised young adults who are generally in good health, the proportion of those reporting gastrointestinal, metabolic, hepatic, or renal diseases was less than 5%. No significant associations were found between halitosis and these self-reported systemic conditions. However, this finding should be interpreted with caution, given the small number of affected individuals and the reliance on self-reported data without confirmation from relevant medical records or diagnoses.

In this study, the OLT was employed to diagnose organoleptic halitosis without the use of instrumental analysis. This method provides an effective, economical, and time-efficient means of assessment that can be easily implemented in school settings, especially in population-based epidemiological studies.^36^ While VSCs are the primary components of halitosis-related gases, other non-sulfur volatile compounds may also play a role in oral malodour. Instruments such as the Halimeter and OralChroma are limited to detecting VSCs, whereas the OLT enables the identification of a broader range of odours. However, it is important to acknowledge the inherent limitations of the cross-sectional study design, as causal inferences cannot be drawn. Some variables, such as stress perception, dry mouth, and awareness of food impaction, were found to be significantly associated with self-reported halitosis, but these associations may be bidirectional. For instance, individuals experiencing halitosis may become more aware of oral discomfort or develop increased psychological stress, rather than these factors necessarily contributing to the onset of halitosis. Longitudinal research is needed to clarify the directionality of these associations. Lastly, this study focuses on young adults, among whom a large proportion had an OS of 1. Although a cutoff of OS ≥2 is commonly used in halitosis research, including studies involving young adults, future investigations may benefit from exploring an intermediate threshold such as OS = 1.5, which could better capture subtler distinctions in younger individuals with heightened odour sensitivity.^7,44^

## CONCLUSIONS

In conclusion, academic pressure, lifestyle changes, and inadequate oral hygiene practices may be associated with halitosis among young adults, particularly university students. Self-reported halitosis is primarily influenced by subjective factors related to self-perception, which differ from those identified through objective testing, such as the OLT. For dental professionals, understanding the aetiology and population-specific correlates of halitosis is crucial for accurate diagnosis and evidence-based management. Moreover, incorporating halitosis prevention and management into school oral health programmes would enhance overall health and well-being.

### Acknowledgements

We sincerely thank all participants from Wuzhou University, Yulin Normal University, Guangxi Science and Technology Normal University, Hechi University, Guangxi Vocational and Technology Institute of Industry, Beibu Gulf University, Guangxi Medical College, Guangxi Normal University for Nationalities, Guangxi University of Science and Technology, and Nanning University of Digital Technology, as well as all volunteers who contributed to this study.

### Funding

This work was supported by the National Natural Science Foundation of China (NSFC) (82060202) and Natural Science Foundation of Guangxi Zhuang Autonomous Region (No. 2025GXNSFAA069622).
